# The high impact of zinc chromium oxide nanocombs on development of larvicidal and antimicrobial performance

**DOI:** 10.1186/s13065-023-01108-9

**Published:** 2024-01-12

**Authors:** Wageha A. Mostafa, Soad A. Elshanawany, Khadejah D. Otaif, Mona khalifa, Elsayed Elgazzar

**Affiliations:** 1 Entomolgy Section, Zoology Department, Faculty of Science, Zagazig University, Zagazig, Egypt; 2 Physics Department, Jazan University, 86736 Jazan, Saudi Arabia; 3 Department of Chemistry, Samtah University College, Jazan University, 86736 Jazan, Saudi Arabia; 4 Biochemistry department, Faculty of Science, Cairo University, Cairo, Egypt; 5 Department of Physics, Faculty of Science, Suez Canal University, Ismailia, 41522 Egypt

**Keywords:** Cr/ZnO nanocombs, Energy gap, Larvicidal, Ultrastructure alteration, Bactericidal

## Abstract

Zinc chromium oxide (Cr/ZnO, 5wt.%) was prepared by a facile chemical co-precipitation route. The structure, composition, and chemical bonding were analyzed using X-ray diffraction (XRD), Energy dispersive X-ray spectroscopy (EDX), and Fourier-transform infrared spectroscopy (FTIR) indicating that chromium ions were integrated the host framework to form Cr/ZnO nanocomposite. Scanning electron microscopy (SEM) and Transmission electron microscopy (TEM) micrographs showed comb-shaped nanoparticles with an average size 20 nm and large surface area. The energy gap of the thin films was estimated from T% and R% measurements which exhibit a strong optical absorption edge close to the visible spectrum. The insecticidal activity of the synthesized nanocombs against *C. pipiens* larvae was evaluated with LC_50_ (30.15 ppm) and LC_90_ (100.22 ppm). Besides, the nanocomposite showed high antibacterial performance against gram-positive bacteria (*Bacillus subtilis*) and gram-negative bacteria (*Proteus vulgaris*) with inhibition zones 21.9 and 19 mm, respectively.

## Introduction

Global warming causes climate variation which is the essential factor in the spread of climate-sensitive infectious [1]. A temperature rise enhances vector and pathogen metabolism allowing faster prevalence, whereas unpredictable rainfall patterns may excess the availability of favorable breeding sites [2, 3]. The combination of warm and moderate humidity provides typical conditions for several types of species *Aedes*, *Culex*, and *Anopheles* mosquitoes to survive transferring diseases like dengue, filariasis, yellow fever, and zika virus [4, 5]. For instance, lymphatic filariasis and West Nile fever are mainly vectored by *Culex pipiens*. Additionally, controlling mosquito vectors carries several dangers due to the vector resistance to conventional pesticides as well as their negative impact on the environment and human health [6, 7]. On the other side, microorganisms including bacteria, fungi, and protozoa pose a great danger to individuals, animals, and plants. World Health Organization (WHO) states that the rapid emergence of infectious diseases owing to infectious bacteria threatens all the world's countries causing millions of deaths annually [8]. Biofilm formation by pathogens in bacterial communities such as multicellular organisms show significant resistance to antibiotics leading to health and economic problems especially in poor countries because of the high therapy costs [9]. Taking these risks seriously, there is an urgent need to uncover economical, biocompatible, competitive, and effective alternative methods to replace traditional treatments.

Nanomaterials have emerged as strong antimicrobial and larvicidal agents depending on their quantum size effect, large surface area to volume ratio, and high chemical reactivity. Metal and metal oxide nanoparticles (NPs) have been applied in various fields comprising medicine, drug delivery, energy, agricultural, catalysis, and biosensors [10]. For example, zinc oxide (ZnO), magnesium oxide (MgO), tin oxide (SnO_2_), silver oxide (Ag_2_O), zirconium oxide (ZrO_2_) and copper oxide (CuO) have been utilized as highly efficient nanomaterials in health care sector [11–13]. In particular, ZnO NPs have drawn much attention as an antimicrobial and anticancer agent for inhibiting the microorganism’s growth thanks to their semiconducting nature, safety, and unique physicochemical characteristics. Recent studies have reported the capability to modify the microstructure of nanomaterial by doping for enhancement of its physical and chemical properties. Integration of the ZnO matrix with different metal ions; Mn, Fe, Cr, Ag, Cu, Cd, Sn, Ga, etc., will create intermediate energy levels and hence increase free charge carriers’ mobility [13, 14]. Tuan Vu et al. have developed gold/zinc oxide (Au/ZnO) nanoflower as a strong photocatalyst and an antimicrobial agent for the degradation of Tartrazine (TA) and killing *Escherichia coli* (E. coli) [15]. Also, Abd El-Latef et al. have substituted Cu^2+^ into ZnO matrix by hydrothermal assisted co-precipitation method for enhancement of insecticidal activity against *Spodoptera littoralis* [16]. Among various transition metals, chromium is a promising dopant owing to the close ionic radius of Cr^3+^ (0.063 nm) to Zn^2+^ (0.074 nm); besides, the electronegativity of Zn is similar to Cr $\sim$ 1.66. Moreover, chromium oxide (Cr_2_O_3_) is a p-type with an energy gap of 3.10 eV which is useful for increasing active sites and oxygen species [17, 18]. In this context, Shah et al. have prepared ZnO and chromium doped ZnO nanoparticles using leaf extract of *Citrus reticulata* indicating strong biological activities against *E. coli*, *S. aureus*, *K. pneumonia* and *P. aeruginosa* with zone of inhibition of 22.4 ± 1.98, 15.5 ± 0.78, 10.2 ± 0.55 and 7.5 ± 1.55 mm, respectively dependence of high generation of reactive oxygen species [19]. In medical applications, Cr doped ZnO has been applied as a therapy for estimating the cytotoxic effects using human fetal lung fibroblast cells (WI38 cells) based on the morphological nature of the nanoparticles [20]. To date, several techniques are employed for fabricating nanoscale materials including hydrothermal, co-precipitation, chemical vapor deposition, spray pyrolysis, solution combustion, sol–gel, and magnetron sputtering. Among them, a simple, scalable, and controllable chemical co-precipitation technique was performed to obtain high quality nanoparticles [20, 21]. The current work aims to modify the topological nature of ZnO NPs by Cr^3+^ replacements to one dimensional nanocomb structure for biomedical applications.

## Experimental section

### Chemicals and reagents

Zinc chloride dihydrate, ZnCl_2_.2H_2_O, 98.0%, Sodium hydroxide (NaOH ≥ 98.0%), Chromium nitrate nonahydrate Cr(NO_3_)0.9H_2_O, ≥ 97.0%), Cacodylate buffer, Glutaraldehyde, Osmium tetroxide (OsO_4_), Ethanol, and Acetone were purchased from Alfa Aesar, All reagents were used without any purification,

### Preparation and characterization of zinc chromium oxide nanocomposite

Zn_x_Cr_1-x_O, x $=$ 5 wt% was prepared by chemical co-precipitation approach. Firstly, 8.35 g zinc chloride dihydrate was dissolved in 50 ml double distilled water and 1.28 g chromium nitrate nanohydrate in 25 ml under a magnetic stirrer. Chromium salt solution was carefully added to zinc chloride with continuous stirring for 3h at room temperature. 2.92 g sodium hydroxide (NaOH) was dissolved in 65 ml double distilled water and then slowly added drop by drop to aqueous solutions under stirring at a constant speed for 4h without heating. A homogeneous white gray precipitate was formed at pH ~ 9. Then separated using filter paper and washed many times with distilled water. The resulting powder was dried in a furnace at 70 °C overnight and eventually annealed at 400 °C for 2h.

### Characterization of the nanomaterial

The phase and crystallographic features of Cr/ZnO nanocomposite were recognized by XRD (Bruker D8 Advance Eco) operated at λ = 1.54 Å(Cu Kα radiation) at 2θ angle varied from 25° to 75°. Morphological nature and elemental composition were recorded using scanning electron micrograph (SEM; Helios Nanolab. 400) attached with energy dispersive X-ray analysis (EDX). The functional group was identified by Fourier transform infrared (FT-IR spectrum 100, Perkin Elmer) at a resolution of 400–2000 cm^−1^ with 16 scan speed. Transmission electron microscopy (TEM; Model: JEOL JEM-2100) was carried out to visualize the average size and particle’s shape. A spin coater sputter coater (Model, EMS 150T ES) was utilized to fabricate the thin films. Transmittance (T%) and reflectance (R%) spectra were measured via UV–Vis spectrophotometer JASCO (V-570) to investigate the energy gap of the fabricated thin films.

### Mosquito rearing

The egg rafts of *C. pipiens* were kept and hatched at a controlled temperature of 27 $-$ 30° and 80% humidity. The larvae fed on fish food and grew in plastic trays that measured 30 $\times$ 25 $\times$ 5 cm. As for the adult mosquitoes, they were kept in cages that measured 30 $\times$ 30 $\times$ 30 cm and fed with a solution that contained 10% glucose.

### Larvicidal activity

The insecticidal activity was investigated by placing 25 mosquito larvae into 200 mL of sterilized double–distilled water with Cr/ZnO at concentrations (25, 50, 100, 150, and 200 ppm) in a 250 mL beaker. Pure water was applied as a control for each individual concentration. The dead larvae were recorded after 24h of treatment, and the death percentage was calculated from the average of replicates [22]. Further, this formula was used to determine the fatal rates.

1 $${\text{Mortality }}\left( \%  \right){\mkern 1mu}  = {\mkern 1mu} \frac{{\left( {No.of{\mkern 1mu} dead{\mkern 1mu} larvae} \right)}}{{\left( {total{\mkern 1mu} of{\mkern 1mu} larvae{\mkern 1mu} \,introduce} \right)}}{\mkern 1mu}  \times {\mkern 1mu} 100$$

### Ultrastructure examination

The midgut ultra-structural alteration of *C. pipiens* larvae was investigated after exposure to Cr/ZnO NPs according to El-Samad et al. with a little modification [23] and the tissues were examined with a JEOL 1200 EX II transmission electron microscope at the central laboratory, Faculty of Science, Zagazig University.

### Antibacterial activity

The antibacterial performance of the prepared specimen was investigated by the disc diffusion method against pathogenic bacteria such as *Staphylococcus aureus*, *Bacillus subtilis* and *Bacillus cereus* (gram-positive), and *Escherichia coli*, *Proteus vulgaris* and *Pseudomonas aeruginosa* (gram-negative). Whatman No.1 filter paper discs of 6 mm dia. were immersed with 10 and 30 μg of Cr/ZnO NPs synthesized nanocomposites for each strain and determined the zones of inhibition around the disks after incubating the plates at 37 °C for 24 h [24]. Each examination was repeated three times, then the data were analyzed using statistical methods and using Gentamycin (4 µg/ml) as a positive control of bacteria. All findings were statistically calculated as the mean ± standard error (SE) of the average values. The chi-square method was utilized to show the significant difference, the obtained data was compared with the control group using regression co-efficient. Also, Probit transformation analysis was applied to determine the LC_50_ and LC_90_ values.

## Results and discussion

### Structural analysis

**Fig. 1:**
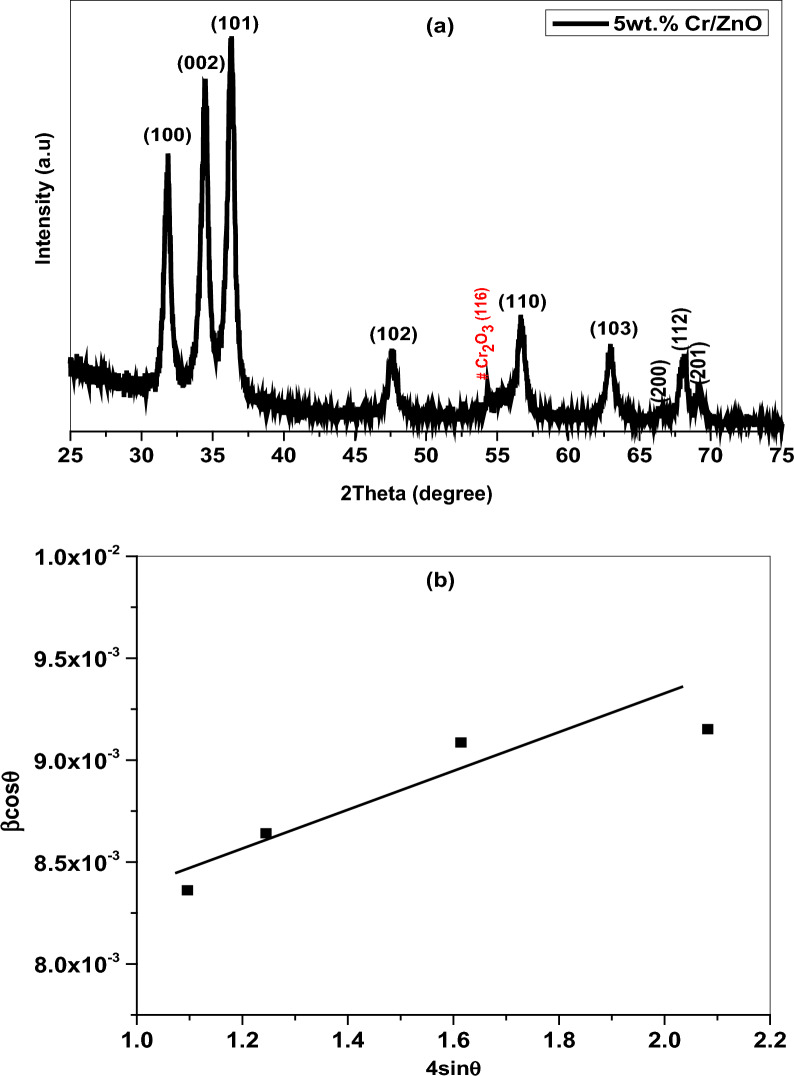
**a** XRD pattern and **b** W–H plot of Cr/ZnO nanocomposite

The XRD profile of Cr/ZnO nanostructures was illustrated in Fig. 1a. As can be seen, the prepared nanopowder of polycrystalline nature and well-defined peaks located at 31.85°, 34.49°, 36.30°, 47.72°, 56.79°, 62.98°, 66.59°, 68.17°, 69.24° corresponding to the reflection planes 100, 002, 101, 102, 110, 103, 200, 112, 201 ascribed to the hexagonal wurtzite structure and coincide with the standard card JCPDS No. 36–1451, space group (P63mc). The sample has a relatively intense peak however the observed broadening clearly reveals the small crystal size of the nanocomposite. No phases related to impurities or unreacted ions were observed in the pattern, otherwise the weak peak located at 2 $\uptheta =$ 54.25° is attributed to chromium oxide (Cr_2_O_3_). Despite the close ionic radius of Cr^3+^ to Zn^2+^, the large amount of chromium ions concentrations (Cr^3+^, 5 wt.%) inside the host framework led to phase segregation [25–27]. Moreover, the crystallographic parameters including crystalline size $({\text{D}})$ and lattice strain $\left(\upvarepsilon \right)$ were determined from the Williamson-Hall (W–H) plot expressed as:

2 $$\mathrm{\beta cos\theta }=\frac{\mathrm{k\lambda }}{{\text{D}}}+4\mathrm{\varepsilon sin\theta }$$

where, $\upbeta$ is the full width at half maximum (FWHM), $\uptheta$ is the Bragg’s diffraction angle, $\uplambda$ is the X-ray wavelength $=$ 1.5418 Å, and k is a constant $=$ 0.94. The internal strain was given from the slope and the crystallite size was defined from the intercept $=\frac{\mathrm{k\lambda }}{{\text{D}}}$, Fig. 1b. Scherrer equation was also applied to evaluate the size of crystals by the following equation:

3 $${\text{D}}=\frac{\mathrm{K\lambda }}{\mathrm{\beta cos\theta }}$$

Lattice strain $(\upvarepsilon )$, dislocation density $(\updelta )$ and degree of crystallinity $\left({{\text{X}}}_{{\text{c}}}\right)$ were estimated by the formulas:

$\upvarepsilon =\frac{\upbeta }{4\mathrm{ tan\theta }}$ $\updelta =\frac{1}{{{\text{D}}}^{2}}$ , and${{\text{X}}}_{{\text{c}}}=\frac{0.24}{\beta }$, respectively. As presented in Table 1, the nanocomposite shows a crystallite size range from 17–19 nm. In addition to, large strain and dislocation density associated with the presence of Cr contents and crystal defects that prevented the crystal growth result in crystal size reduction [27, 28].

**Table 1:** Crystallographic parameters of Cr doped ZnO nanocomb obtained from W–H plot and Scherrer formula

Crystallographic parameters						
W–H plot			Scherrer equation			
Nanocomb	${\text{D}}$(nm)	$\upvarepsilon \times {10}^{-4}$	${\text{D}}$(nm)	$\upvarepsilon \times {10}^{-4}$	$\updelta \times {10}^{-4}{\mathrm{ nm}}^{-2}$	${{\text{X}}}_{{\text{c}}}$
Cr/ZnO	18.97	7.84	16.74	69.00	35.70	26.73

### Composition and functional group identification

**Fig. 2:**
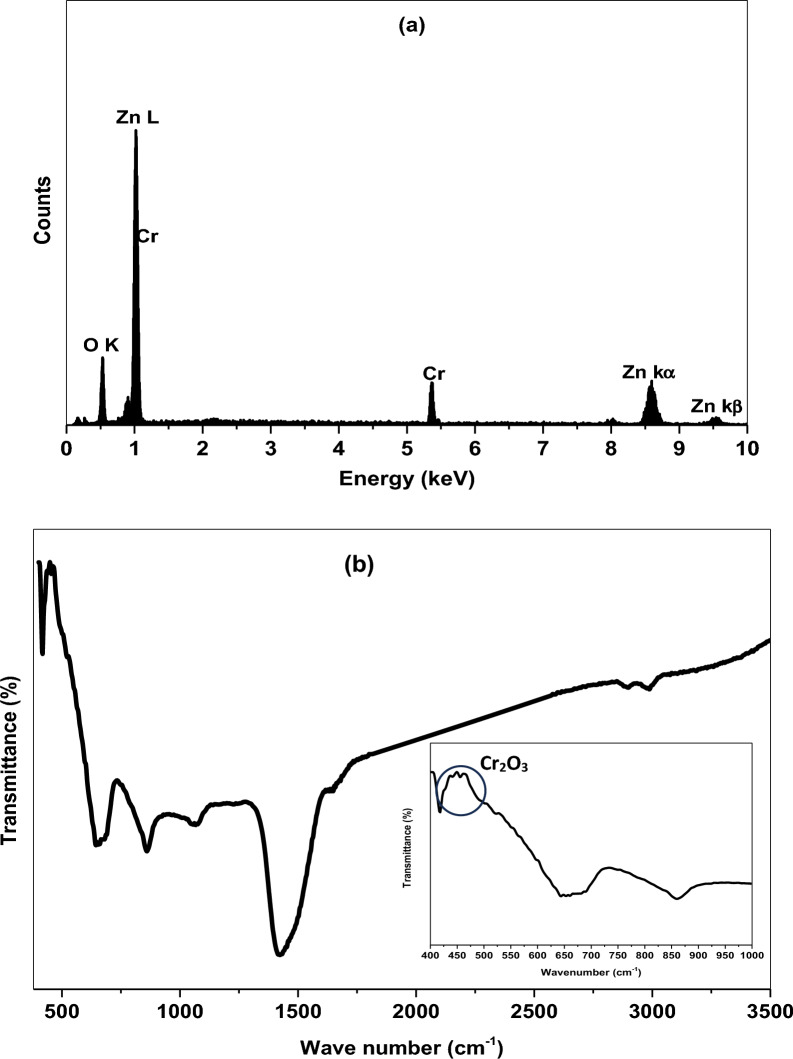
**a** EDX spectrum and **b** FTIR of Cr/ZnO nanocomposite

The chemical composition and purity of Cr/ZnO NPs were investigated from EDX spectroscopy. The spectrum has detected the compositional peaks of Zn, O, Cr elements of weight percentages (wt.%) 62.16, 29.72, and 8.12 respectively. The presence of chromium peak in EDX spectrum reveals the incorporation of Cr^3+^ into the host matrix, Fig. 2a [29, 30]. The absence of impurities related to unreacted ions emphasizes the high purity of the nanocomposite. The functional group and chemical bonding were defined from FTIR through wavenumber range from 400 to 3500 cm^−1^ as depicted in Fig. 2b. The vibrational bands at 729.43 cm^−1^ and 860.52 cm^−1^ are attributed to the stretching mode the Zn $-$ O bond. Besides, the weak absorption bands at 438.40 cm^−1^ is assigned to chromium oxide (Cr_2_O_3_). The two weak bands at 447.67 cm^−1^ and 462.23 cm^−1^ are associated with metal oxide Zn $-$ O $-$ Cr and Cr $-$ O bonds, respectively. In general, nanometal oxides exhibit vibrational frequencies at wavenumber less than 1000 cm^−1^. The observed weak IR bands at wavenumber varied between 1265 and 1470 cm^−1^ are assigned to O-H stretching vibrations which arises from absorbing atmospheric moisture [31, 32]. The FTIR spectrum clearly approves that chromium ions have occupied zinc oxide framework as demonstrated in the XRD result.

### Morphological characteristics

**Fig. 3:**
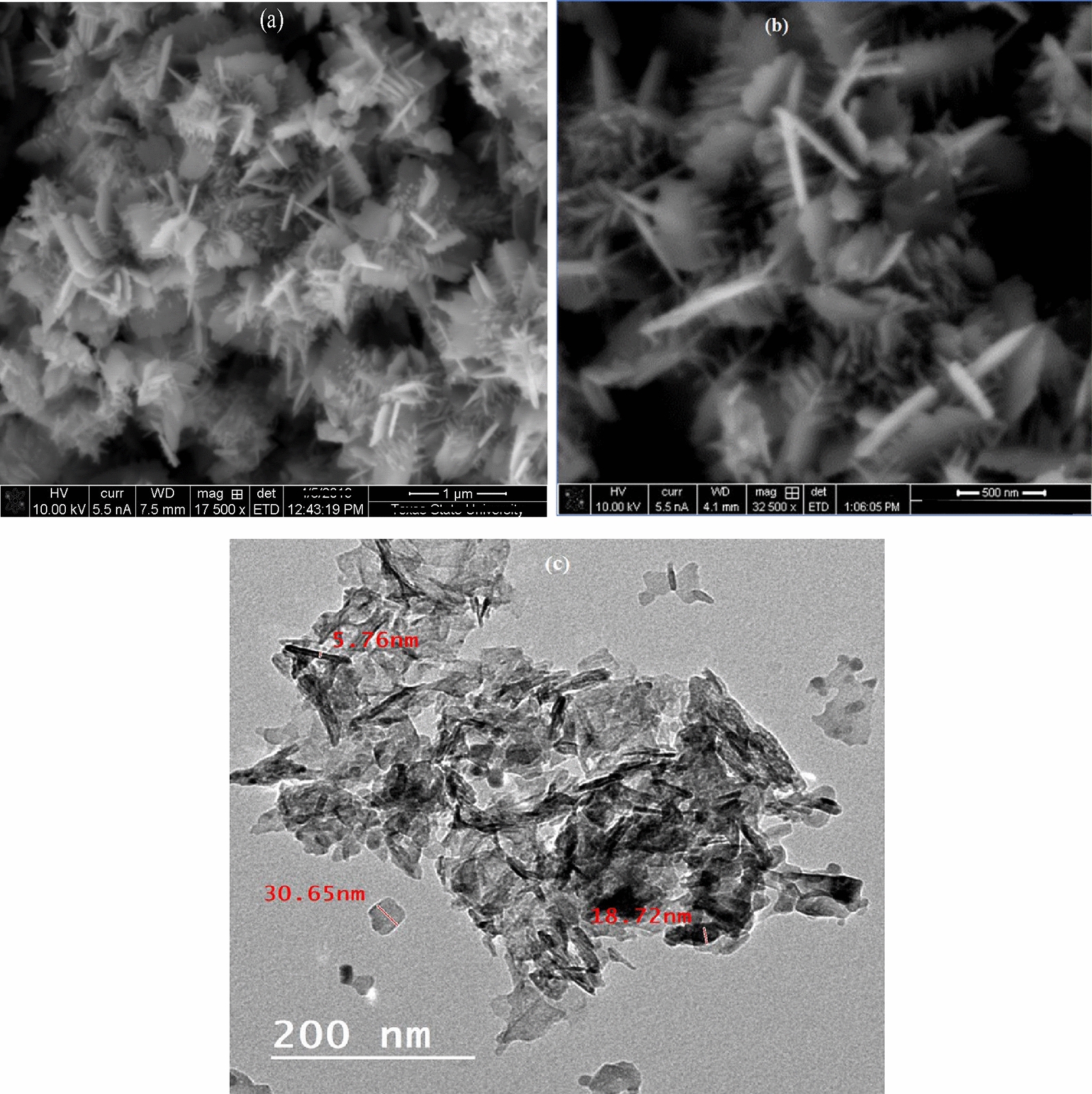
**a, b** SEM micrograph and **c** TEM micrograph of Cr/ZnO nanocomb

The surface morphology of the synthesized nanocomposite was visualized by SEM micrograph executed at different magnifications. As described in Fig. 3(a,b), the particles appear in nanocomb-like shapes, aggregated together with high density. The surface appears not uniform and has different shapes; some particles take thin flake shape [33]. Sharp needles linked to nanocomb were observed in the SEM image, Fig. 3b. These needles have a strong impact on killing microbes and mosquitoes. Further, average size and particles distribution were determined by TEM micrograph. Figure 3c, shows the particles in thin sheet, needle, and spherical shape with mean size $\sim$ 20 nm. Little needles are assembled in nanorods of diameter $\sim$ 6 nm, length 80 nm, and aspect ratio $\sim$ 13 [34, 35]. The microstructure analysis has approved that the nanocombs possess superior architecture topological features. Where, the dimensions confinement and small size enable them to be used as antimicrobial and larvicidal agent for human health protection.

### Optical analysis

**Fig. 4:**
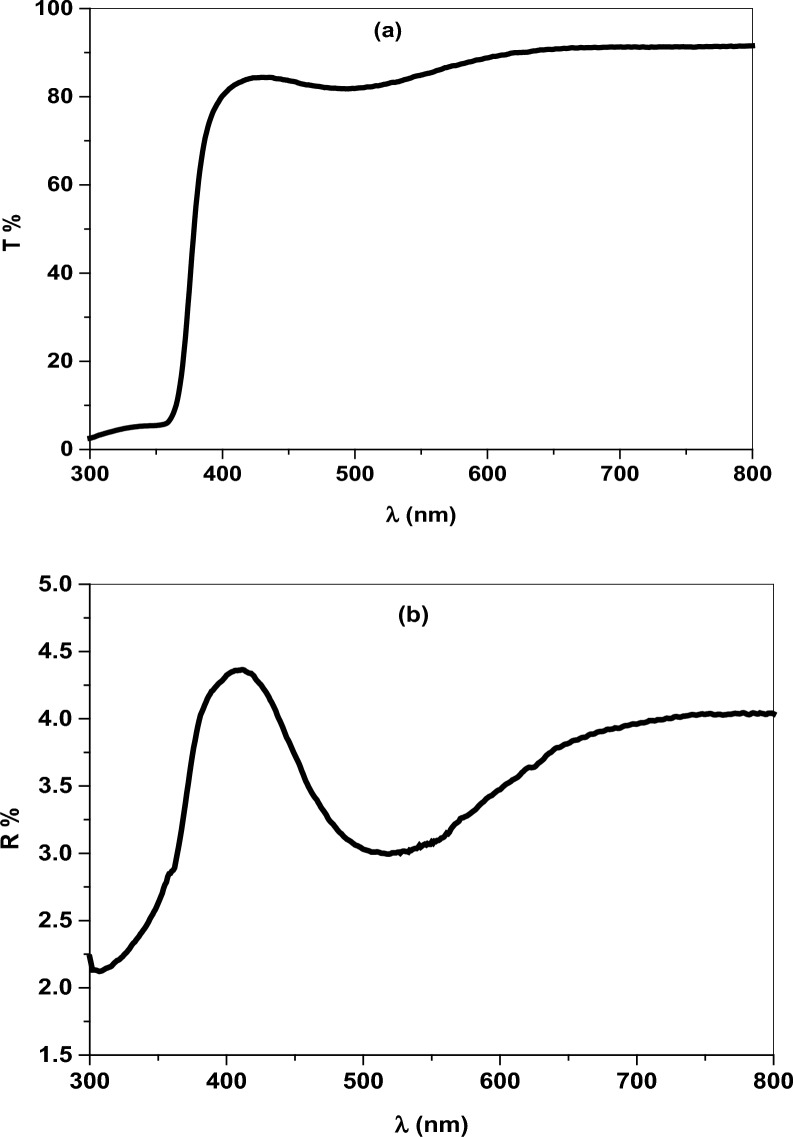
**a** Optical transmittance and **b** Reflectance of Cr/ZnO thin film

The optical characteristics of Cr/ZnO coated thin film were analyzed from transmittance (T%) and reflectance (R%) spectra through the wavelength varied between 300 $-$ 800 nm. The thin film exhibited high optical transmittance greater than 82% in the visible region. Besides, a strong absorption edge was detected at 390 nm attributed to electron transition from valence band (V.B) to conduction band (C.B), Fig. 4a. The optical reflectance displayed peaks and valleys ascribed to electron-photon interaction or electron scattering, Fig. 4b [36, 37]. The optical absorption coefficient ($\mathrm{\alpha })$ was calculated from T% and R% using the following relation:

4 $$ \alpha = \frac{1}{d}Ln\left[ {\frac{{(1 - R^{2} )}}{2T}\,\, + \sqrt {\frac{(1 - R)}{{4T^{2} }}\, + \,R^{2} } } \right] $$

**Fig. 5:**
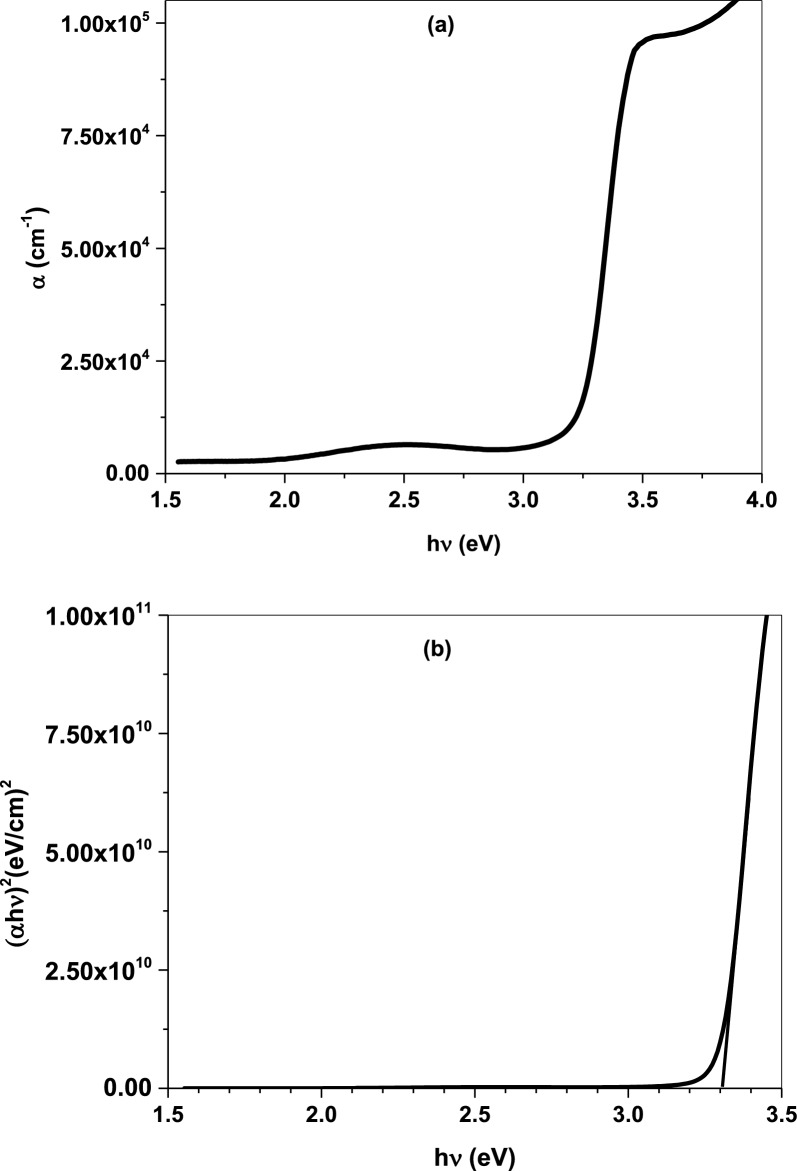
**a** Optical absorption and **b** Energy gap of Cr/ZnO thin film

Figure 5a illustrates the absorption coefficient as a function of photon energy $(\mathrm{h\nu })$. The thin film exhibits an optical absorption peak at $\sim$ 3.20 eV in which the electron absorbs incident photon energy and jumps to the conduction band leading to increased free charge carriers [36]. For further optical analysis, the optical band gap $({{\text{E}}}_{{\text{g}}})$ was determined from Tauc’s equation expressed as [37]:

5 $$\mathrm{\alpha }=\frac{{\text{A}}}{\mathrm{h\nu }} (\mathrm{h\nu }-{{\text{E}}}_{{\text{g}}}{)}^{{\text{n}}}$$

Here, A is an independent constant, and n is constant value determines the electron transition behavior. here, n was taken by the value 0.5 refers to allowed direct transition. The ${{\text{E}}}_{{\text{g}}}$ of Cr doped ZnO was evaluated from ${(\mathrm{\alpha h\nu })}^{2}$ versus $(\mathrm{h\nu })$ plot by extending the straight part to zero photon energy axis equals 3.31 eV, Fig. 5b. The wide energy gap emphasizes the quantum size effect of the nanocomposite which is attributed to the large surface area and reactive surface sites [37, 38]. Cr/ZnO nanocombs show a wide energy gap that is attributed to the small particle size. The substitution of Cr in the host lattice results in broadening of the energy gap, which also generates more free charge carriers according to Moss-Burstein effect. Both reactive oxygen species (ROS) in addition to metal ions production have a strong impact on harmful internal components comprising proteins and DNA causing bacterial and larvicidal death [39].

### Mosquito larvicidal activity

**Fig. 6:**
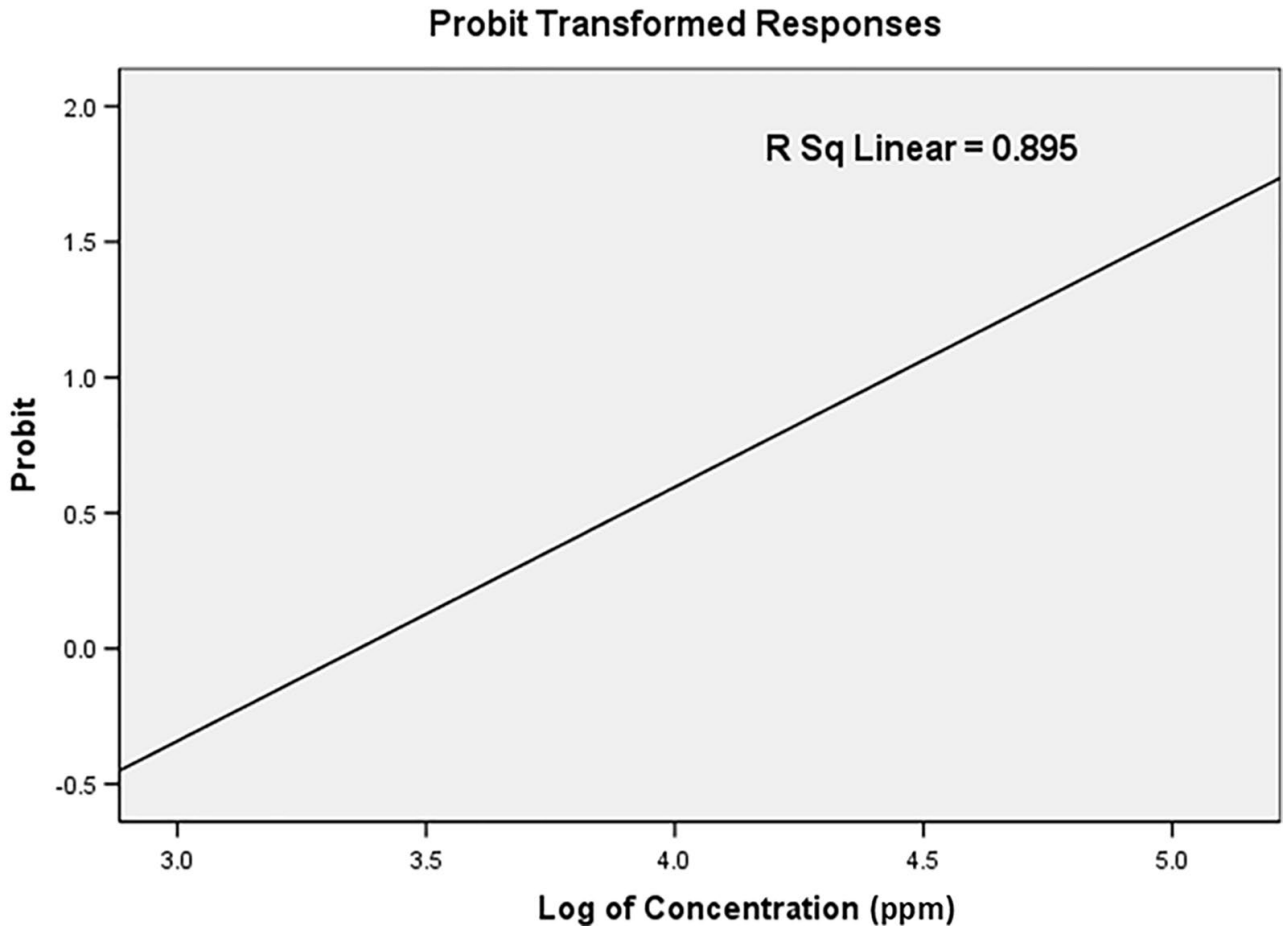
The probability analysis of mortality of *C. pipiens* mosquito larvae exposed to Cr/ZnO nanocomb

Different concentrations of Cr/ZnO nanocomb were applied on the third larval instars of *C. pipiens* for 24 h to evaluate their efficacy. The findings refer to an increase in nanoparticle concentrations leading to high larval mortality. As presented in Table 2, the death rate was 100% at the ratio of 200 ppm and 35 ± 0.629% at 25 ppm. The LC_50_ and LC_90_ values were 30.15 and 100.22 ppm, respectively while the control did not show any toxicity to mosquito larvae and Chi-square (χ2) value shows a significant difference at p < 0.01 level. The probability analysis of *C. pipiens* larval mortality utilizing the nanocomposites was shown in Fig. 6. The results of this work indicated the mortality of mosquito larvae caused by the internal toxic power of small particles inside the cuticle and the accumulation of nanoparticles in the alimentary canal particularly in the midgut which represents a defense region against the toxicity of insecticides. Also, comb and needle particles can penetrate the larval midgut, damaging DNA, overlapping with organelles, and interrupting physiological processes, which prevent mosquito larvae growth and cause death [40, 41]. Related results were reported by Gunathilaka et al. and Ibrahim and Ali, ZnO NPs were effective against mosquito larvae caused mortality to the first larval instar of *C. pipiens* and star-shaped ZnO NPs were more effective against larvae than needle, round, and cubical-shapes [42, 43]*.* Moreover, metal and metal oxide nanoparticles have strong toxicity and physiological impact on insect pests [43–45].

**Table 2:** Toxic effect of nanostructure Cr/ZnO against third larval instar of *C. pipiens*

Concentrations (ppm)	Mean ± S.E	LC_50_	LC_90_	Regression equation	X^2^
		(LCL)	(UCL)		
25	35 ± 0.629^a^				
50	73.75 ± 0.47^ab^	30.15	100.22	Y = -4.07 + (X × 1.19)	9.7
100	80 ± 0.64^b^	(24.12: 35.71)	(82.55:131.66)		
150	92.5 ± 0.288^c^				
200	100 ± 0.00^d^				

LC_50_ and LC_90_: lethal concentration that kills 50 and 90% of the exposed *C. pipiens* larvae; UCL: upper confidence limit; LCL: lower confidence limit; X^2^ = Chi-square, p < 0.05, significance level, Mean value of five replicates, (SE) standard error. Control (distilled water), nil mortality. Letters indicate degree of significant based on Tukey’s HSD tests between different treatments and control. Letter (a) mean high significant, and letter (c) mean low significant, while the same letters are not significantly different

### Light and SEM images

**Fig.7:**
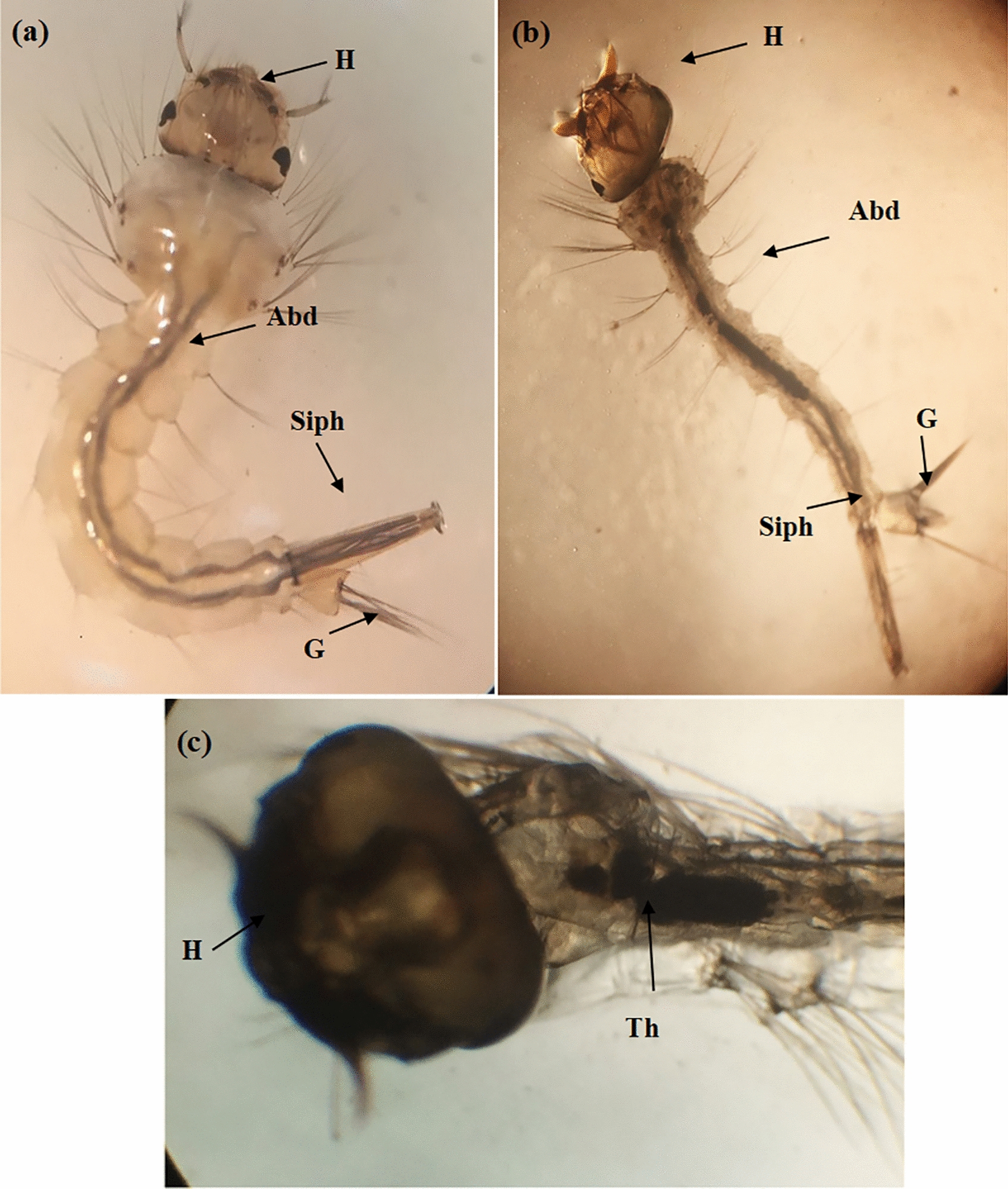
Light microscope photographs of body parts (head, abdomen, and respiratory opening) of the 3rd larval instars of *Cx. pipiens* control **a** and larvae treated with Cr/ZnO NPs **b**, **c** (10 × 40) (Abd: abdomen; G: gills; H: head: Siph: siphon: Th; thorax)

**Fig. 8:**
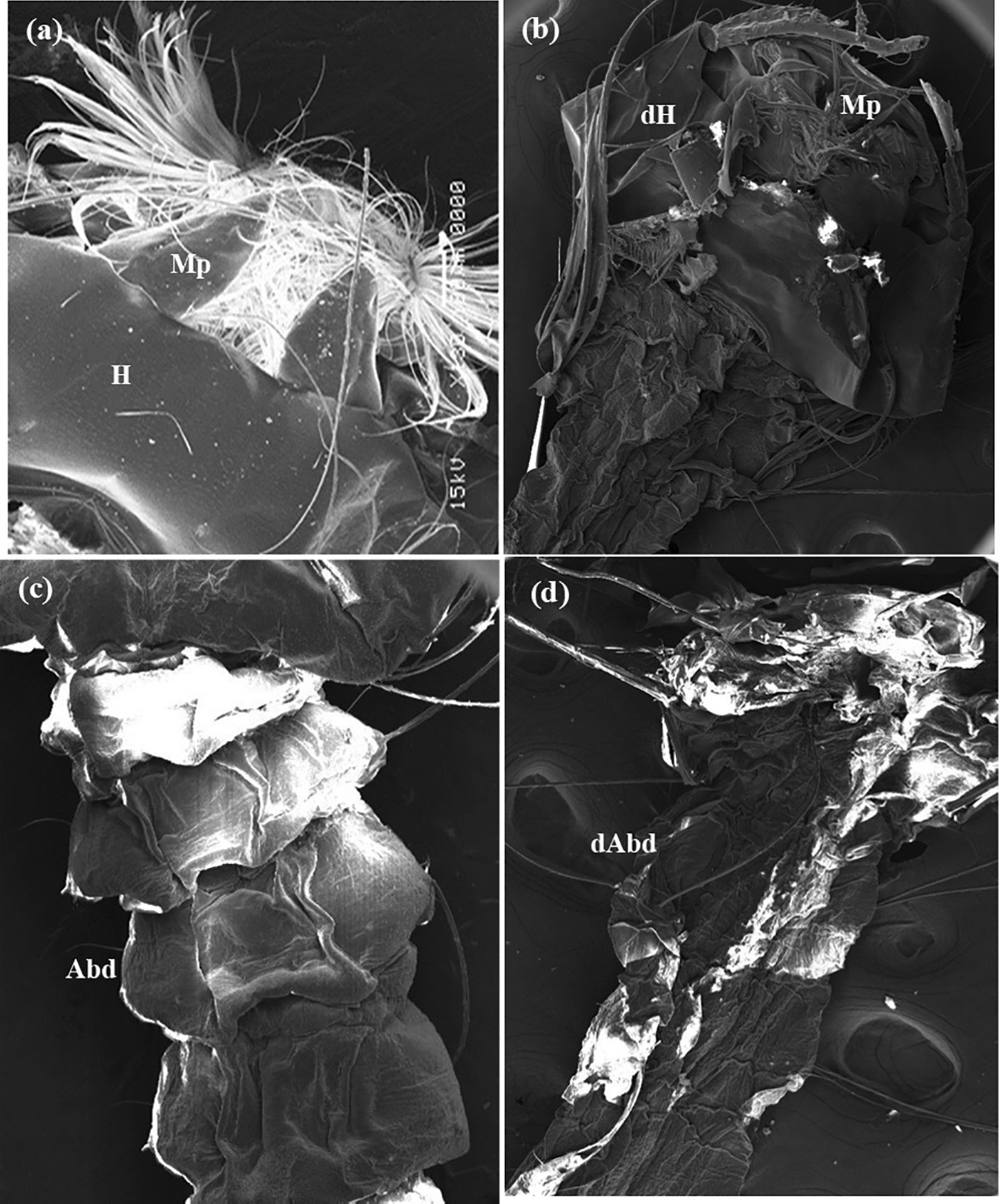
The SEM micrographs of third larval instar of control *C. pipiens* larvae **a**, **c** showed normal head capsule, mouth parts and abdominal segments, **b**, **d** larvae treated by Cr/ZnO NPs at 48 h post treatment showed deformed head capsule, mouth parts and abdominal segments (*Abd* abdomen, *dAbd* deformed abdominal segments, *H* head: d H: deformed head: Mp; mouthparts)

The synthesized Cr/ZnO nanoparticles impacting *C. pipiens* larvae caused a change in their morphology features as indicated by histopathological studies after 48 h of treatment, as illustrated in (Figs. 7, 8). Light microscope images of treated larvae with fabricated samples showed the deformation in head and abdominal segments with an accumulation of nanoparticles in the alimentary canal (Fig. 7b, c) compared with the normal structure of the controlled one (Fig. 7a). Moreover, SEM microscopy images of the control larval instar observed normal morphological structures of the head and abdominal structure without any malformation (Fig. 8a, c), while (Fig. 8b, d) illustrated the destroyed head and mouth parts and deformation of the abdominal segments. This observation is similar to the works of literature [46, 47].

### Ultrastructural changes in the midgut of *C. pipiens* larvae by Cr/ZnO treatment

**Fig. 9:**
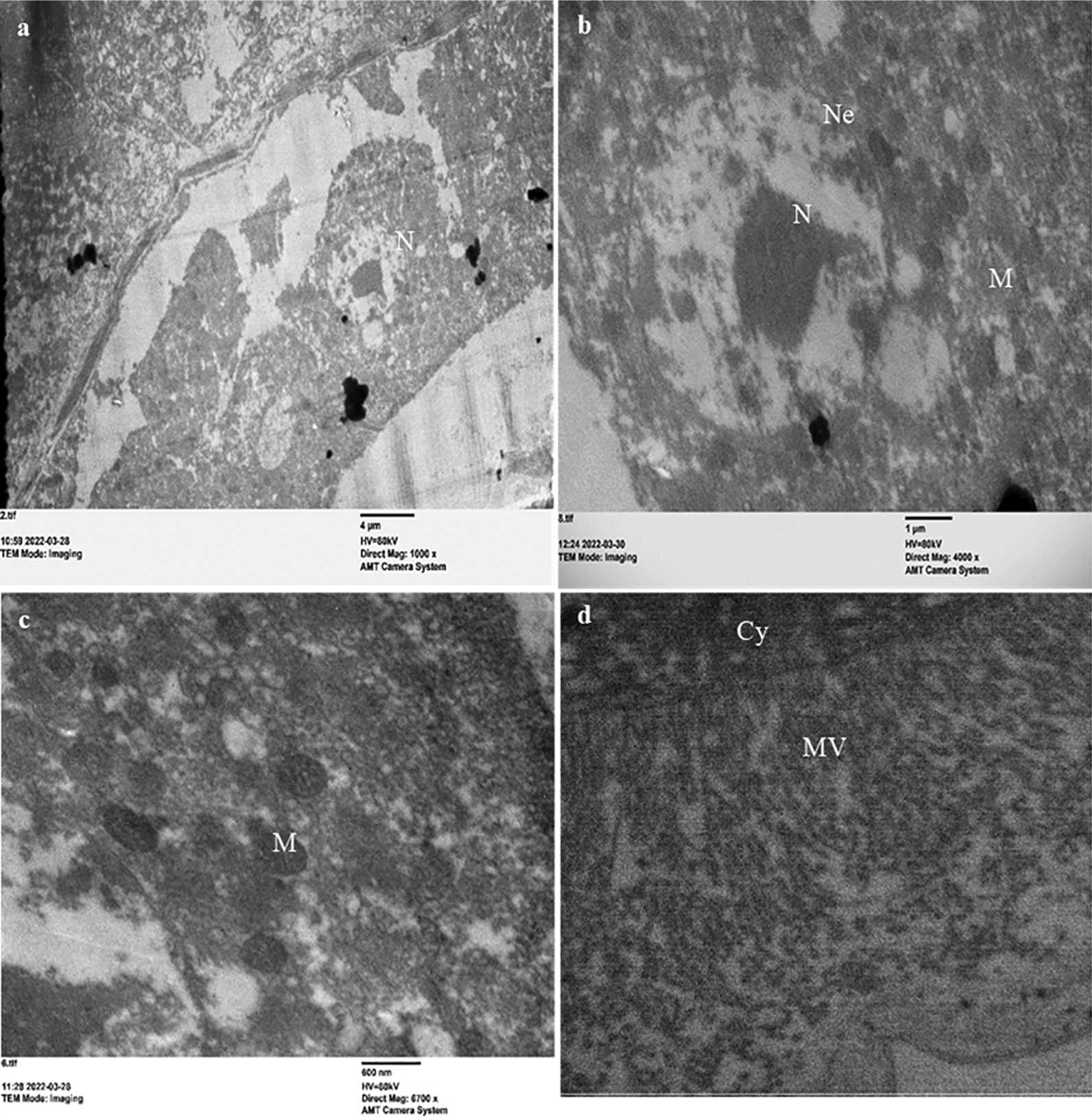
Transmission electron micrographs showing the ultrastructural in the midgut of the normal third larval instar of *C. pipiens* mosquito **a**, **b** The normal structure of the epithelium showing the nucleus having scattered chromatin. **c**, **d** the gut cytoplasm has mitochondria and microvilli. Cy(cytoplasm), M (mitochondria), N (nucleus), Ne (nuclear envelope), and MV (microvilli)

**Fig. 10:**
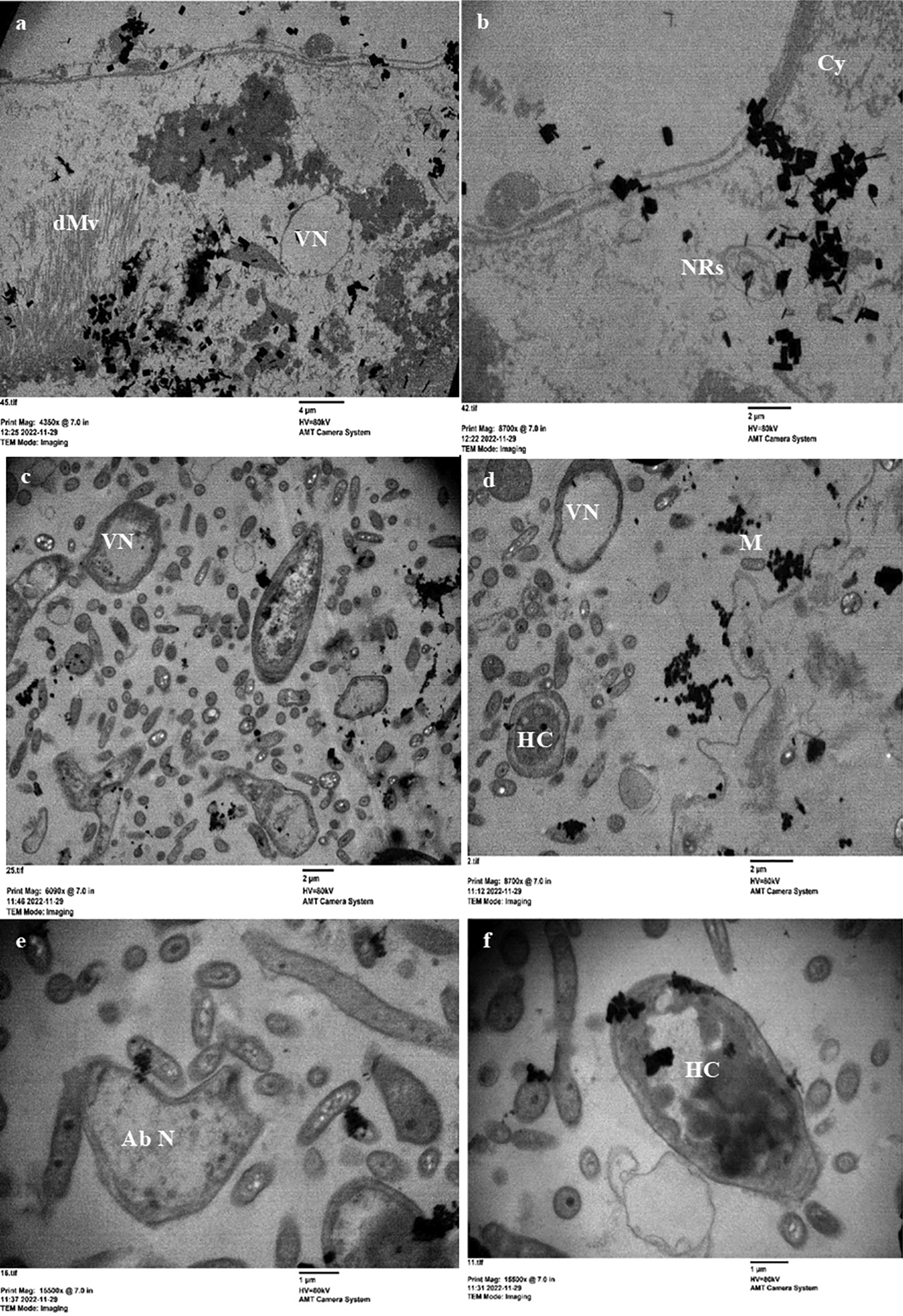
Transmission electron micrographs showing the ultrastructural changes in the midgut of the third larval instar of *C. pipiens* mosquito in response to LC_50_ treatment with Cr/ZnO NPs **a**, **b** An epithelial cell in treated larvae showing missed microvilli and clear condensation of the nucleus with clear vacuolation in the cytoplasm near the basement membrane. **c**, **d** the cells have vacuolated nuclei and nucleus with heterochromatin. **e**, **f** The epithelium in the treated larva vacuolated and have abnormal nucleus shapes. Cy (cytoplasm), dMV (microvilli), HC (heterochromatin), M (mitochondria), Ab N (abnormal nucleus) and VN (vacuole nucleus)

Transmission electron micrographs of the midgut of control and LC_50_ treated *C. pipiens* larvae illustrated how the particles interact with the larval body cells compared to the control larvae. The ultrastructure of the normal epithelial cell of *C. pipiens* larvae midgut has uniform microvilli, and the nucleus with scattered chromatin (Fig. 9a, b) was depicted previously by literature [48]. The cytoplasm of the control larvae showed regular structure and normal distribution of the cell organelles and normal nuclei are central and rounded as shown in (Fig. 9c, d). Conversely, Cr/ZnO nanocomb treated midgut epithelial cells display complete destruction and rupture near the basement membrane. There was a clear abnormal nuclei shape with heterochromatin and the aggregation of a high amount of needle-shape nanocomposites in the cytoplasm destroyed cell structure and entered the hemocoel resulting in insect death. (Fig. 10a, b, c). Moreover, the treated larvae showed several forms of vacuolation in the cytoplasm, and a clear condensation appeared in the nucleus and vacuolated nuclei (Fig. 10d, e, f). Similar changes have been reported by Ibrahim et al. about the abnormal appearance of the midgut epithelia as condensation of the nuclear chromatin, abnormal shape of nuclei, absence of microvilli, and appearance of numerous vacuoles of *C. pipens* immature stages exposed to ZnO NPs [49]. The gut in insects has a key role in regulating different mechanisms as a vital organ in ecotoxicological investigations since the epithelial midgut is the first organ exposed to a large amount of toxins ingested by an insect [50]. Additionally, the midgut represented a physical and chemical barrier to toxic substances ingested during feeding and ZnO NPs treated larvae showed that the microvilli of the midgut cells lining were very short compared to control as well as many vacuolations appeared in the cytoplasm and a clear condensation appeared in the nucleus [51]. Mohamed et al. reported that MnCoO nanostructures induce significant damage in the midgut structure of *C. pipiens* which showed the destruction of columnar epithelial cells causing the widening of intercellular spaces, and cytoplasmic vacuolization [52]. The mode action of metal oxide nanoparticles in the larval midgut via its influence on digestive tract enzymes, DNA malformation, production of reactive oxygen species, causing cellular leakage, and disorder of the organelle functions causing larval death [53].

### Antimicrobial activity

**Fig. 11:**
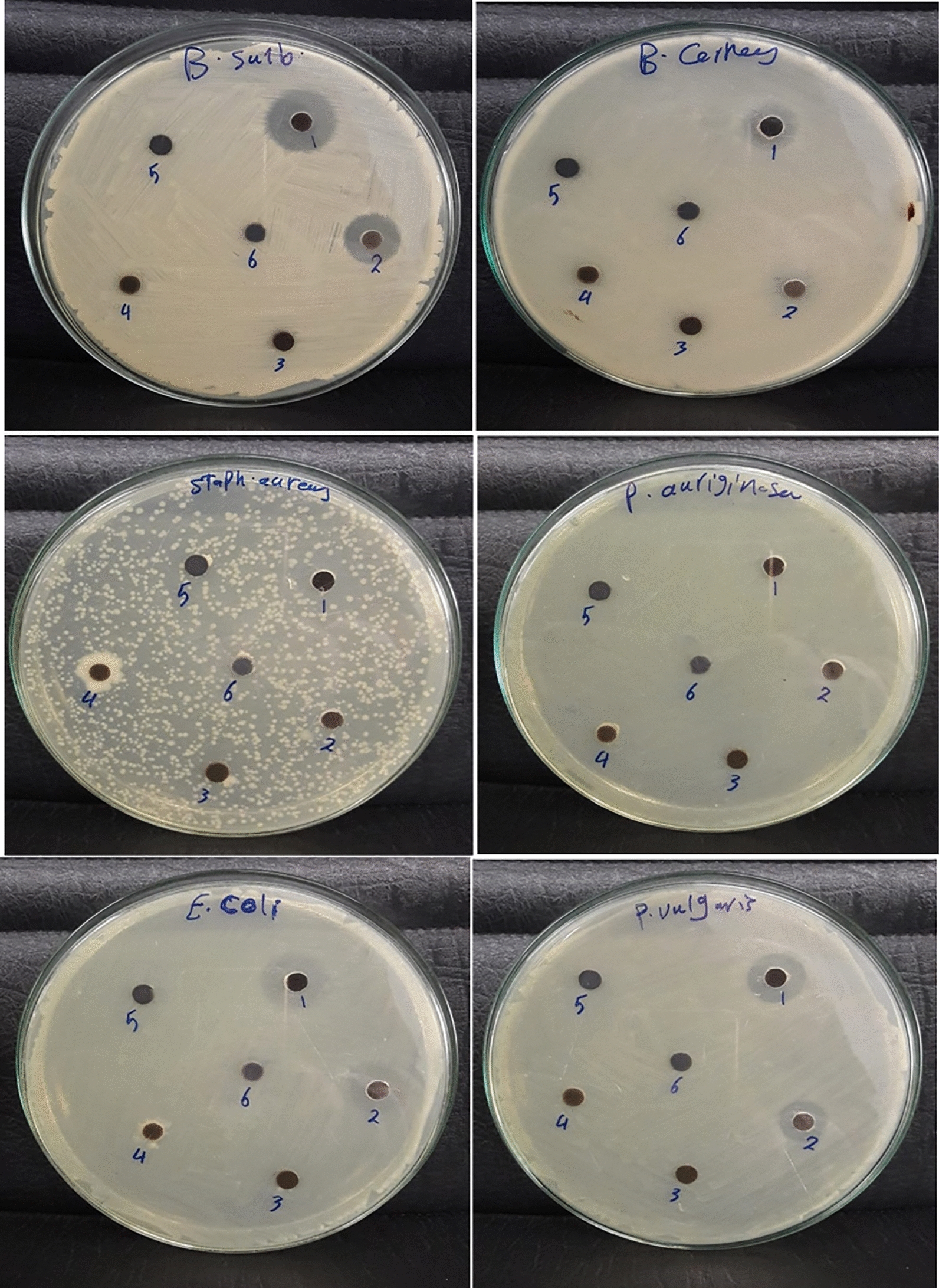
The inhibition zones indicating the antimicrobial activity of Cr/ZnO against gram positive and gram-negative bacteria

**Fig. 12:**
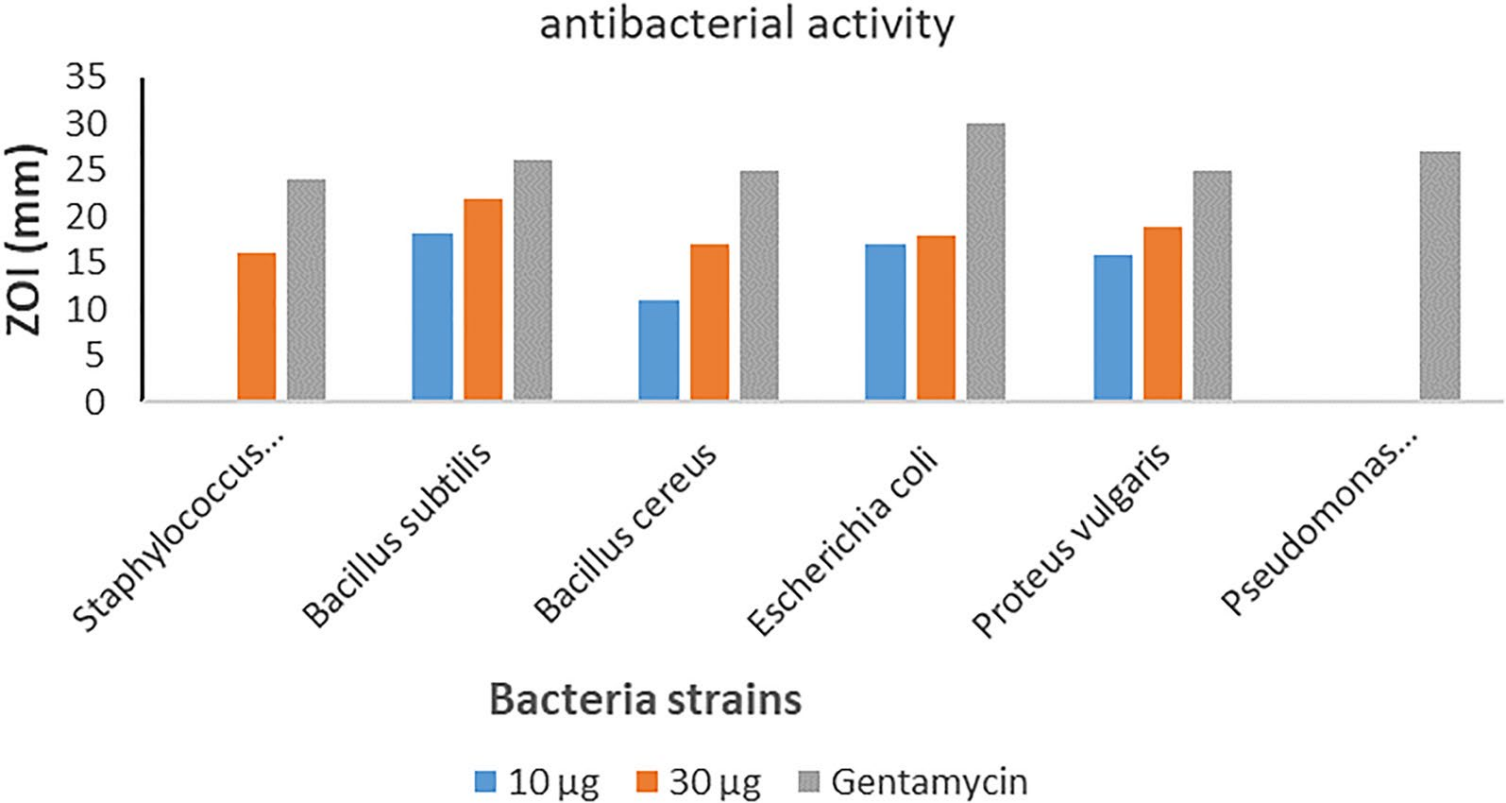
A chart depicting zones of inhibition of Cr/ZnO NPs against the test bacteria

Antimicrobial results revealed that the Cr doped zinc oxide nanoparticles are more efficient against pathogenic bacteria at high concentrations. As shown in (Fig. 11, 12) and Table 3, *Bacillus subtilis* was the most sensitive among the tested microbes with 21.9 mm inhibition zone at 30 µg. Moreover, the inhibition zones of *Staphylococcus aureus* and *Bacillus cereus* were 16 and 16.93 mm, respectively at the high concentration*. Escherichia coli* and *Proteus vulgaris* have an inhibition zone of 17.93 and 19 mm, respectively while *Pseudomonas aeruginosa* showed non sensitivity at the same concentration. However, the control drug exhibited higher activity compared to Cr/ZnO nanocomb as demonstrated in the Table 3. From the results, the antibacterial activity of the nanoparticles was concentration dependent. The comb shape of Cr doped ZnO increased the penetration and harmed both gram-positive and gram-negative bacteria as the needles produced from it enhanced the potential activity of nanocomposite in destroying the pathogenic bacteria although most bacterial structures are resistant to major antibiotics. Besides that, Gram-negative bacteria have a great attraction towards positively charged nanoparticles, which result in cellular destruction [54]. In the present work, the antimicrobial activity of the nanocomb is efficacious against pathogenic microorganisms. These findings agree with earlier studies that demonstrated the antibacterial potential of Cr doped ZnO nanostructure against pathogenic gram- positive and gram-negative bacteria such as *Escherichia coli*, *Staphylococcus aureus*, *Pseudomonas aeruginosa*, and *Bacillus cereus* [55, 56]. Also, Vijayalakshmi and Sivaraj investigated the antibacterial activity of Cr doped ZnO nanocomb results indicated enhanced bactericidal activities against *E. coli* and *S. aureus* compared to undoped ZnO–NPs [57]. The enhanced bactericidal activity of Cr-doped ZnO was associated with an increase in ROS production, and a reduction in particle size [58, 59].

**Table 3:** Antibacterial activity of Cr/ZnO nanocomb

Inhibition zone diameter in mm	Cr/ZnO (Mean ± SE)		Positive control
Tested bacteria	10 µg	30 µg	
Gram positive bacteria			Gentamycin
* Staphylococcus aureus*	Non sensitive	16 ± 0.45	24 ± 1.07
* Bacillus subtilis*	18.13 ± 0.41	21.9 ± 0.85	26 ± 0.64
* Bacillus cereus*	11 ± 1.oo	16.93 ± 0.60	25 ± 1.04
Gram negative bacteria			
* Escherichia coli*	16.96 ± 0.25	17.93 ± 0.60	30 ± 0.20
* Proteus vulgaris*	15.96 ± 0.45	19 ± 1.00	25 ± 1.09
* Pseudomonas aeruginosa*	Non sensitive	Non sensitive	27 ± 0.86

**Fig. 13:**
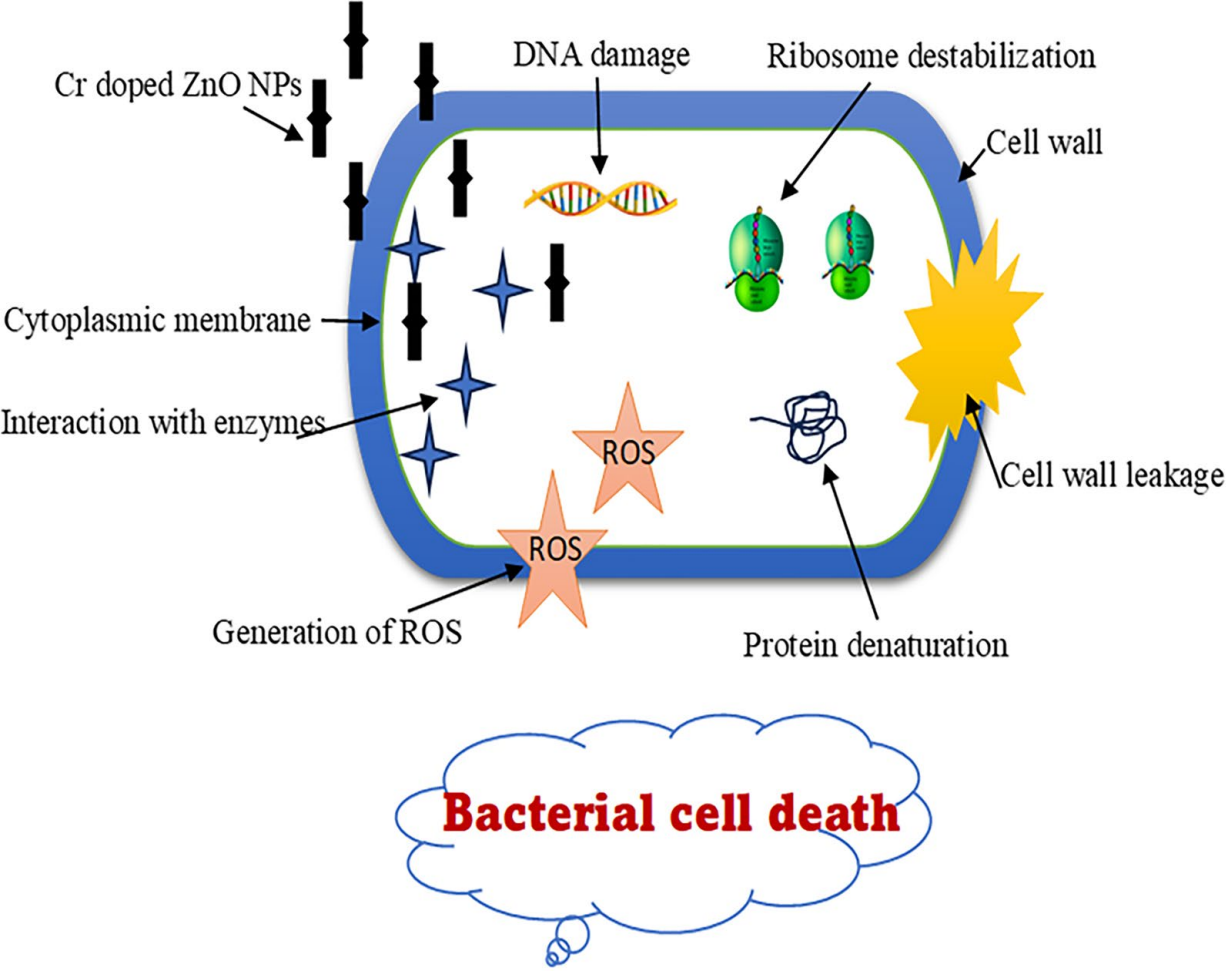
Antibacterial activity mechanism of Cr/ZnO NPs

Furthermore, the mechanisms of metal oxide nanomaterials as anti-bacterial agents show through interrupting the microbial cell directly or producing reactive oxygen species (ROS) that cause DNA damage, lipid peroxidation, and protein oxidation, also can destroy bacterial cells via the penetration and leakage of the bacterial membranes [60–62]. These intracellular functional disorders are initiated by the oxidative stress manipulated by ROS leading to cell death as illustrated in Fig. 13 according to Shah et al. [54, 60]. These results coincided with Olejnik et al. (2021) who reported that the ZnO nanocomb recorded higher toxicity as compared with spherical particles underlining the significance of particle size and shape for cytotoxicity [63]. Also, Babayevska et al. (2022) have shown that zinc oxide rods have the ability to penetrate through bacteria more easily than spheres [64]. Moreover, the Cr doped ZnO provides a more effective anti-bacterial agent compared to the pristine ZnO [57].

## Conclusions

Cr doped ZnO nanocomposite was successfully synthesized by simple co-precipitation method. The prepared sample was characterized using various spectroscopic techniques suggesting small crystallite size, large internal strain, and dislocation density of $\sim 36\times {10}^{-4} {{\text{nm}}}^{-2}$. Integration of Cr^3+^ inside the host ZnO matrix was confirmed using EDX and FTIR spectra. The SEM images revealed the nanocomb structure of Cr/ZnO with aspect ratio $=$ 13 and large surface area. Moreover, the TEM image showed the particles in nanoflakes linked with sharp needles. The morphological analysis emphasized the special shape and structure of the nanocomposite which can enable it to be used as an efficient larvicidal and anti-bacterial agent. The fabricated thin film exhibited a wide band gap of 3.31 eV close to the visible region. In larvicidal study, the nanoparticles result in 100% mortality at concentration 200 ppm beside that the treated larvae showed destruction in midgut epithelium cells and malformation of their organelles. In antimicrobial activity, the nanocomb strongly caused DNA damage to bacterial cells and cell membrane leakage which results in cell death. In conclusion, the synthesized Cr/ZnO nanocomposite exhibited a significant impact on eradicating microbes and mosquitoes anchored to their superior structural-morphological and optical characteristics.

## Data Availability

The datasets used and/or analyzed during the current study are available from the corresponding author on reasonable request.

## Abbreviations

NPs: Nanoparticles
XRD: X-ray diffraction
EDX: Energy dispersive X-ray spectroscopy
FTIR: Fourier-transform infrared spectroscopy
SEM: Scane electron microscopy
TEM: Transmission electron microscopy
Abd: Abdomen
G: Gills
H: Head
Siph: Siphon
Th: Thorax
dAbd: Deformed abdomin
dH: Deformed head
Mp: Mouthparts
Cy: Cytoplasm
M: Mitochondria
N: Nucleus
Ne: Nuclear envelope
MV: Microvilli.
Nu: Nucleus
Ch: Chromatin clumps
dMV: Microvilli
HC: Heterochromatin
Ab N: Abnormal nucleus
VN: Vacuole nucleus
